# Decolorization and detoxication of plant-based proteins using hydrogen peroxide and catalase

**DOI:** 10.1038/s41598-022-26883-8

**Published:** 2022-12-27

**Authors:** Kiyota Sakai, Masamichi Okada, Shotaro Yamaguchi

**Affiliations:** Amano Enzyme Inc., Innovation Center, Kakamigahara, Japan

**Keywords:** Enzymes, Proteins, Industrial microbiology, Applied microbiology

## Abstract

The gap between the current supply of meat and its predicted future demand is widening, increasing the need to produce plant-based meat analogs. Despite ongoing technical developments, one of the unresolved challenges of plant-based meat analogs is to safely and effectively decolor plant proteins that originally exhibit yellow–brown or strong brown color. This study aimed to develop an effective and safe decoloring system for soy-based protein products using food-grade hydrogen peroxide and catalase. First, soy-based protein isolate (PI) and textured vegetable protein (TVP) were treated with hydrogen peroxide, and then the residual hydrogen peroxide was degraded using catalase. This process caused notable decolorization of PI and TVP, and residual hydrogen peroxide was not detected in these products. These findings indicate that this process could safely and effectively decolorize soy-based proteins. Interestingly, this decoloring process enhanced the solubility, water- and oil-holding capacities, foaming capacity, and emulsifying stability of decolored soy-based PI. Additionally, cooking loss and juiciness of decolored TVP-based foods were improved compared to those of non-treated foods. These findings indicate that the decoloring process also enhances the physical properties of soy-based protein products.

## Introduction

The global population is expected to reach 9.7 billion by 2050, and this is forecasted to lead to a greater increase in the demand for dietary proteins^1^. The widening gap between the supply and demand for meat or fish, as the primary source of dietary protein, has increased the need to produce plant-based foods^2^. Additionally, it has been reported that adopting a plant-based diet has significant health benefits^3^. Numerous studies have reported health benefits associated with the replacement of animal sources of meat with plant-based meats, including reduced risks of type 2 diabetes, heart disease, and stroke^4,5^. Moreover, reducing fish and seafood consumption may prevent the intake of toxic substances, such as heavy metals and mercury^6–8^. Therefore, developing better plant-based diets would address the current protein crisis and positively impact human health^2^.

Despite ongoing technical developments, the appearance, flavor, taste, and texture of plant-based meat analogs (e.g., meat and seafood) differ from those of animal-based products. One of the unresolved challenges for plant-based meat analogs is their appearance, particularly their color^9^. Color is the first aspect of food products noticed by consumers and is a major contributor to consumers’ perception of taste and overall product acceptance^10^. Most protein isolate (PI) products are yellow–brown to strongly brown in color. Therefore, red pigments (e.g., betanin and anthocyanin) are added to red plant-based foods, such as meat (e.g., patties and sausages) and seafood (e.g., tuna and salmon) analogs. However, these red pigments cannot completely mask the strong brown color of plant-based proteins. Moreover, white plant-based seafood analogs (e.g., cod and squid) and dairy (e.g., cheese and yogurt) turn brown owing to the presence of plant-based proteins. These unacceptable color changes affect the consumer acceptance of plant-based products and remain a critical obstacle^11^. Therefore, many companies and researchers have focused on decolorizing plant proteins.

PIs are traditionally manufactured through alkali extraction with heating, centrifugation, and subsequent isoelectric point precipitation^12^. Textured vegetable proteins (TVPs) that imitate the fibrillar structure of meat muscles are produced from PIs using an extruder^2^. The Maillard reaction in these two manufacturing processes can lead to an undesired color and flavor formation in plant-based foods^13^. The main coloration of plant-based proteins is the formation of melanoidins on the furan backbone^14–16^. This protein coloration is an irreversible condensation reaction between the carbonyl groups of melanoidins and the amino groups of lysine and arginine^17^. In addition, a previous study reported that removing phenolic compounds leads to a slight decolorization of soy proteins^18^. Therefore, phenolic compounds with hydrophobic interactions with plant-based proteins are also considered the cause of coloring.

Various chemical and physical processes, such as activated carbon adsorption^19,20^, the organic solvent method^21,22^, exchange resin adsorption^23^, and hydrogen peroxide oxidation^24^, are used for decolorization. Among these, the decolorization of plant-based proteins using activated carbon adsorption has been extensively studied^25^. However, the activated carbon adsorption method has some shortcomings^25^. Primarily, separating fine-particle activated carbon from a protein solution is difficult. Further, the process is not selective and leads to high protein loss. Decolorization by hydrogen peroxide oxidation is a promising strategy because of its low cost. Additionally, regulatory authorities have already permitted the use of hydrogen peroxide for various purposes, such as antimicrobial agent in starch and milk for cheese-making and as a decolorization agent in beef, tripe, and instant tea^26^. However, despite the increasing demand for decolorized proteins, the decolorization, characterization, and changes in the nutritional value of meat analog products and protein ingredients, such as soy-based PI and TVP, using hydrogen peroxide have not been widely examined^24^.

Regulatory authorities require the complete removal of hydrogen peroxide from final food products^26^. Therefore, in this study, we used catalase (CAT) to degrade residual hydrogen peroxide after decolorizing plant-based proteins. CAT (EC 1.11.1.6) catalyzes the decomposition of hydrogen peroxide into water and oxygen^27^. This enzyme has been applied in a wide range of industrial applications such as textile decolorization^28^, cheese production^29^, and prevention of lipid oxidation in meat^30^. To our knowledge, methods for decoloring plant-derived proteins using hydrogen peroxide followed by detoxification with CAT enzyme have not been evaluated till date. This study aimed to develop a more effective and safe decoloring method using hydrogen peroxide and a catalase detoxification method for plant-based proteins. The physical and nutritional properties of decolorized soy-based PIs and TVPs were also characterized.

## Results and discussion

### Biochemical characterization of food-grade CAT

**Figure 1:**
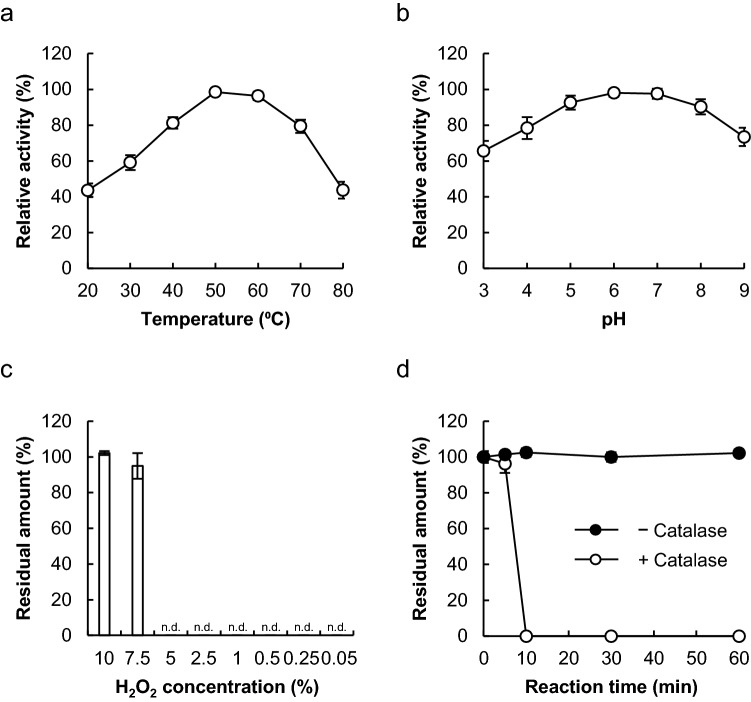
Biochemical characterization of food-grade catalase (CAT). (**a**) Optimal temperature for catalase (CAT) activity. Enzyme reactions proceeded at temperatures ranging from 20 to 80 °C. (**b**) Optimal pH for CAT activity. Enzyme reactions proceeded over a pH range of 3.0–9.0 in 100 mM McIlvaine buffer. (**c**) Hydrogen peroxide stability of CAT. Substrate hydrogen peroxide concentrations ranged from 0.05 to 10%. (**d**) Time-course of degrading hydrogen peroxide by CAT. Error bars represent the mean ± standard error of the mean of three independent experiments.

We used catalase NP “Amano” 5, a commercially available food-grade CAT, to detoxify hydrogen peroxide. We first investigated the optimal temperature and pH for CAT to remove hydrogen peroxide. The optimal temperature for CAT was 50 °C, with a preferred temperature range (> 80%) between 40 and 70 °C (Fig. 1a). The optimal pH was 6.0, with a preferred pH range (> 80%) of 5.0 and 8.0 (Fig. 1b). We next investigated the maximum concentration of hydrogen peroxide that could be detoxified using CAT, as higher concentrations of hydrogen peroxide typically deactivate enzymes. CAT completely degraded 5% and less hydrogen peroxide in 10 min (Fig. 1c,d).

### Color analysis of decolored soy-based PI and TVP

**Figure 2:**
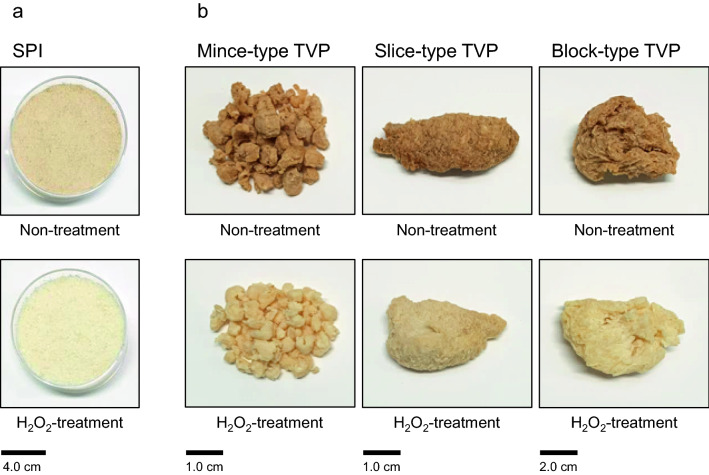
Appearance of decolored soy-based protein isolate (SPI) and textured vegetable protein (TVP). SPI (**a**) and TVP (**b**) were decolored with hydrogen peroxide, and the residual hydrogen peroxide was degraded by catalase.

To investigate whether hydrogen peroxide can decolor plant-based proteins, soy-based PI and TVP were treated with hydrogen peroxide, and then the residual hydrogen peroxide was degraded by CAT. After CAT treatment, no hydrogen peroxide was detected in the protein solutions. Figure 2 shows the appearance of the SPI and TVP that underwent the process. This process with hydrogen peroxide and CAT visually caused notable decolorization. To investigate the color change in greater detail, the objective color attributes of the proteins were characterized using the L*a*b* coordinates (Table 1). The L* value (lightness) of SPI treated with hydrogen peroxide and CAT was higher than that of untreated SPI. The a* value (redness) of SPI treated with hydrogen peroxide and CAT was lower than that of untreated SPI. Hydrogen peroxide and CAT treatments effectively decolorized various types of TVPs. Therefore, these findings indicate that the decolorization process with hydrogen peroxide and CAT can safely and effectively decolorize soy-based proteins.

**Table 1:** Objective color measurements of decolored soy-based PI and TVP.

Treatment	Protein	L*	a*	b*
Non-treated	SPI	62.4 ± 1.3^a^	4.3 ± 0.3^a^	14.4 ± 2.5^a^
Decolored		78.3 ± 2.1^b^	1.0 ± 0.3^b^	13.9 ± 3.1^a^
Non-treated	Mince-type TVP	53.0 ± 1.5^a^	5.1 ± 0.9^a^	14.5 ± 2.1^a^
Decolored		73.3 ± 2.3^b^	1.1 ± 0.4^b^	13.6 ± 1.5^a^
Non-treated	Slice-type TVP	52.7 ± 1.8^a^	5.2 ± 0.9^a^	12.7 ± 2.6^a^
Decolored		78.0 ± 1.5^b^	1.8 ± 0.1^b^	15.0 ± 0.8^a^
Non-treated	Block-type TVP	54.9 ± 2.5^a^	5.4 ± 0.7^a^	13.9 ± 1.7^a^
Decolored		77.4 ± 1.1^b^	0.9 ± 0.4^b^	15.2 ± 1.1^a^

Data are presented as the mean ± standard deviation of values from three experiments. SPI, soy-based protein isolate; TVP, textured vegetable protein, L*, lightness; a*, redness; b*, yellowness.

The undesirable colors of plant-based proteins are considered to be mainly due to low-molecular-weight melanoidins and minor phenolic compounds^17,18^. The irreversible condensation reaction between the carbonyl groups of melanoidins and amino groups of lysine and arginine is the primary contributor to the protein color^17^. These melanoidins are based on the furan backbone^14,15,31^. A previous study reported that hydrogen peroxide converted furan-2-carbaldehyde into succinic acid, maleic acid, fumaric acid, formic acid, and cinnamic acid^32^. Similarly, another study reported that furfural was decomposed into maleic acid by hydrogen peroxide^33^. These previous studies indicate that hydrogen peroxide could ring-cleave furan rings responsible for exhibiting yellow and brown to black. Thus, it is assumed that hydrogen peroxide could similarly cleave the furan ring of melanoidins which renders the yellow–brown color to the plant-based meats, leading to the decolorization of soy-based PI and TVP.

Phenolic compounds that hydrophobically interact with soy proteins also contribute to the color of plant-based proteins^18^. A previous study reported that removing phenolic compounds led to slight decoloring of soy proteins^18^. Therefore, we determined the total phenolic content in soy protein. Non-treated and decolored soy-based PI had concentrations of phenolic compounds equivalent to 192.6 and 55.6 mg gallic acid equivalents (GAE)/100 g, respectively. These findings indicate that hydrogen peroxide treatment reduced the levels of phenolic compounds in soy-based PI. This suggests that the reduction in phenolic compounds might also have contributed to soy protein decolorization.

### Physical properties of decolored proteins

We investigated the adverse effects of hydrogen peroxide on industrially important protein functionalities such as the water- and oil-holding capacities, foaming properties, and emulsifying properties. These physical properties strongly depend on the water solubility of the protein^34^. Therefore, the degree of hydrolysis and water solubility of decolored proteins were examined. As shown in Table 2, the decolored and untreated proteins were not hydrolyzed. Yao et al. also reported that hydrogen peroxide enhanced *Camellia oleifera* seed protein solubility but did not alter the degree of hydrolysis^12^. Our results showed that the solubility of decolored proteins was improved by 1.77-fold compared to that of the non-treated proteins. In addition, SDS-PAGE showed that hydrogen peroxide treatment increased the amounts of soluble proteins and further formed protein–protein crosslinks (Supplementary Fig. S2). This is because hydrogen peroxide can partly transform amino groups into carbonyl groups in proteins, thereby forming Schiff bases from carbonyl groups to promote covalent crosslinking between different proteins^12,35^. The removal of phenolic compounds by hydrogen peroxide was previously suggested to enhance protein solubility^12^. The interactions between proteins and phenolic compounds denature the proteins, thereby reducing their solubility^36–38^. In agreement with these results, we found that hydrogen peroxide treatment reduced the levels of total phenolic compounds in soy protein. Thus, removing phenolic compounds by hydrogen peroxide might improve protein solubility.

**Table 2:** Physical properties of decolored soy-based protein isolate.

	Non-treated protein	Decolored protein
Degree of hydrolysis (%)	n.d.	n.d.
Solubility (%)	23.37 ± 1.73^a^	41.27 ± 2.20^b^
Water-holding capacity (g/g-protein)	2.83 ± 0.21^a^	4.17 ± 0.31^b^
Oil-holding capacity (g/g-protein)	4.67 ± 0.15^a^	5.67 ± 0.21^b^
Foaming capacity (%)	23.33 ± 4.16^a^	36.67 ± 3.06^b^
Foam stability (%)	95.14 ± 1.49^a^	96.58 ± 0.86^a^
Emulsifying activity index (m^2^/g)	29.54 ± 1.08^a^	32.43 ± 0.55^a^
Emulsifying stability index (%)	14.63 ± 0.91^a^	20.22 ± 2.60^b^

Data are presented as the mean ± standard deviation of values from three experiments.

We investigated various physical properties of the decolored proteins (Table 2). Water- and oil-holding capacities are important factors in the juiciness of plant-based foods. Interestingly, the water- and oil-holding capacities of decolored proteins were significantly higher than those of untreated proteins. Protein foaming properties are important in the processing of creamy plant-based dairy products. The foaming capacity of decolored proteins was also significantly higher, while the foam stability of decolored proteins was similar to that of untreated proteins. Emulsifying properties are important factors for suppressing oil separation and improving the mouthfeel. The emulsifying activity of decolored proteins was also slightly enhanced (P > 0.05), and emulsifying stability was significantly improved compared with that of non-treated proteins (P < 0.05). These findings indicate that hydrogen peroxide and CAT treatment of PI contributed to the industrially important functionalities of plant-based foods.

Previous studies reported that hydrogen peroxide treatment increases the emulsifying and foaming properties of proteins^24,39–41^ This is because hydrogen peroxide treatment expands the protein tertiary structure and exposes some hydrophobic aromatic and aliphatic amino acid side chain groups inside the molecule in polar solutions^24,39–41^. It has been reported that the removal of phenols by macroporous resin also leads to improved emulsification^7,12,42^. This is because the presence of phenolic compounds enhances protein aggregation and reduce the emulsifying ability of proteins^12^. Therefore, improving the emulsifying and foaming activities of decolored soy proteins were considered to be due to the expansion of protein tertiary structures and the removal of phenolic compounds by hydrogen peroxide.

### In vitro digestion of decolored proteins

Hydrogen peroxide is a strong oxidizing agent and, thus, may cause not only protein decolorization but also chemical modification of amino acid residues in proteins. A previous study showed that hydrogen peroxide treatment did not alter the amino acid composition of *Camellia oleifera* seed protein^24^, suggesting that hydrogen peroxide treatment did not reduce the nutritional value of proteins. However, this evaluation did not accurately reveal the amounts of amino acids that were digested and incorporated into the human body. Thus, we investigated the amounts of free amino acids produced from the decolored protein during the gastric and intestinal phases using in vitro digestion (INFOGEST method). The amounts of free amino acids produced from non-treated and decolored proteins upon treatment with digestive enzymes were measured using HPLC. Table 3 shows that the quantities and qualities of amino acids from the decolored protein were similar to those of the non-treated protein. These results indicate that hydrogen peroxide treatment did not adversely affect the bioavailability of amino acids from the digested proteins.

**Table 3:** Amino acid levels at the gastric and intestinal phases during in vitro digestion.

Amino acid (μM)	Non-treated protein		Decolored protein	
	Gastric phase	Intestinal phase	Gastric phase	Intestinal phase
Ala	0.57 ± 0.28^a^	1.84 ± 0.31^a^	0.78 ± 0.33^a^	2.11 ± 0.90^a^
Arg	1.47 ± 0.67^a^	5.89 ± 0.19^a^	1.09 ± 0.21^a^	6.70 ± 1.69^a^
Asn	0.43 ± 0.33^a^	1.10 ± 0.08^a^	0.38 ± 0.24^a^	1.33 ± 0.26^a^
Asp	0.79 ± 0.06^a^	1.05 ± 0.28^a^	0.72 ± 0.11^a^	1.22 ± 0.45^a^
Gln	0.11 ± 0.07^a^	1.99 ± 0.16^a^	0.09 ± 0.10^a^	2.22 ± 0.40^a^
Glu	1.09 ± 0.45^a^	1.11 ± 0.09^a^	1.18 ± 0.05^a^	1.28 ± 0.56^a^
Gly	3.06 ± 0.43^a^	3.22 ± 0.04^a^	3.11 ± 0.16^a^	3.43 ± 0.52^a^
His*	0.22 ± 0.15^a^	0.68 ± 0.05^a^	0.14 ± 0.06^a^	0.73 ± 0.13^a^
Ile*	0.08 ± 0.08^a^	1.92 ± 0.41^a^	0.15 ± 0.10^a^	1.68 ± 0.10^a^
Leu*	1.20 ± 0.11^a^	5.54 ± 0.50^a^	1.46 ± 0.24^a^	6.22 ± 0.92^a^
Lys*	0.25 ± 0.09^a^	3.51 ± 0.19^a^	0.18 ± 0.06^a^	3.70 ± 0.74^a^
Met*	6.99 ± 0.06^a^	7.67 ± 0.08^a^	7.44 ± 0.27^a^	8.22 ± 0.37^a^
Phe*	1.18 ± 0.84^a^	3.92 ± 0.76 ^a^	2.40 ± 0.37^a^	4.49 ± 1.16^a^
Ser	0.43 ± 0.17^a^	1.04 ± 0.09^a^	0.37 ± 0.10^a^	1.16 ± 0.36^a^
Thr*	0.41 ± 0.23^a^	0.97 ± 0.16^a^	0.33 ± 0.13^a^	0.79 ± 0.11^a^
Trp*	0.36 ± 0.28^a^	0.95 ± 0.29^a^	0.31 ± 0.14^a^	1.19 ± 0.60^a^
Tyr	0.33 ± 0.14^a^	3.01 ± 0.25^a^	0.57 ± 0.35^a^	3.30 ± 0.67^a^
Val*	2.27 ± 0.01^a^	2.32 ± 0.10^a^	2.24 ± 0.33^a^	2.53 ± 0.63^a^
Total	21.5 ± 3.85^a^	47.5 ± 3.20^a^	23.2 ± 2.76^a^	51.7 ± 9.12^a^

*Essential amino acids. Data are presented as the mean ± standard deviation of values from three experiments.

### Color and physical properties of decolored TVP-based foods

**Figure 3:**
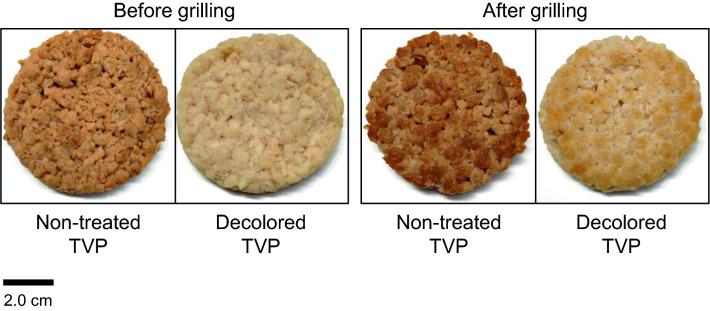
Appearance of decolored textured vegetable protein (TVP)-based foods before and after grilling.

We investigated the color and physical properties of plant-based foods produced from decolored TVP. First, the effects of the grilling process on recoloring of decolored TVP-based foods were measured using a colorimeter (Fig. 3 and Table 4). After grilling process, the brown tone of non-treated TVP-based foods notably became stronger, whereas that of the decolored TVP-based foods changed only slightly. Compared with that of the uncooked mince-type TVP (Table 1), the L* value of non-treated TVP-based foods was low, whereas that of decolored TVP-based foods was higher. These findings indicate that decolored TVP-based foods maintain their white color after the grilling process.

**Table 4:** Objective color measurements and physical properties of decolored textured vegetable protein-based foods.

	Non-treated protein	Decolored protein
L*	50.9 ± 1.9^a^	79.2 ± 1.2^b^
a*	6.7 ± 0.6^a^	3.0 ± 0.3^b^
b*	11.9 ± 1.4^a^	12.5 ± 0.5^a^
Hardness (N)	13.2 ± 0.5^a^	9.8 ± 0.3^b^
Cohesiveness	0.71 ± 0.04^a^	0.83 ± 0.04^b^
Springiness	0.76 ± 0.02^a^	0.85 ± 0.03^b^
Chewiness (N)	7.1 ± 0.6^a^	6.9 ± 0.5^a^
Cooking loss (%)	13.5 ± 1.4^a^	9.6 ± 0.9^b^

Data are presented as the mean ± standard deviation of values from three experiments. L*, lightness; a*, redness; b*, yellowness.

The improved cooking loss of TVP-based foods (Table 4) may be related to the enhanced water- and oil-holding capacities of SPI following hydrogen peroxide treatment (Table 2). Previous studies using IR spectroscopy reported that hydrogen peroxide treatment expanded the tertiary structure of proteins and weakened the intramolecular and intermolecular hydrogen bonds between proteins^24^. Thus, more water molecules may have the opportunity to enter the protein and form new hydrogen bonds with hydroxyl groups in the proteins^24^. This may increase the water-holding capacity of decolored proteins. In addition, several studies have reported that hydrogen peroxide treatment exposes the hydrophobic regions of proteins^39,40^. The partially exposed hydrophobic groups of the protein can interact with lipid molecules^24,39,40^. These effects increase the oil-holding capacity of decolored proteins. Thus, the water- and oil-holding capacities of decolored soy proteins were increased because of the expansion of protein tertiary structures, which led to the formation of new hydrogen bonds with water molecules and hydrophobic interactions with oil molecules. Hydrogen peroxide treatment may similarly enhance the cooking loss of TVP by improving the water- and oil-holding capacities.

The L* values of decolored TVP-based foods (Table 4) were higher than those of decolored TVP (Table 2). Previous studies have reported that L* values increased with increasing water/oil content of plant-based meat analog patties^14,43–46^. This is because small globules such as water or oil cause more light reflection^45,46^. Thus, it is suggested that decolored TVP with higher water- and oil-holding capacity decreased cooking loss, resulting in an increased L* value of decolored TVP-based foods.

## Conclusions

We developed an effective strategy using hydrogen peroxide to decolorize plant-based meat analogs and subsequently detoxify hydrogen peroxide using a food-grade CAT enzyme. Our strategy for decolorizing plant-based meat analogs may enhance the visual impact of plant-based meat analogs and thus improve consumer acceptance. Additionally, this decolorization strategy could improve the quality of plant-based protein analogs by increasing their consumer satisfaction potential.

## Methods

### Materials

Granule-, slice-, and block-type soy-based TVPs were purchased from Marukome Co., Ltd. (Nagano, Japan). CAT (Amano Enzyme, Inc., Nagoya, Japan) is a commercially available food-grade CAT enzyme.

### Enzyme assay

CAT activity was evaluated in 1.0-mL reaction mixtures containing 100 mM McIlvaine buffer (pH 3.0–9.0) and 5.0% (w/v) hydrogen peroxide at 20–80 °C for 10 min. The reaction was stopped by incubation at 100 °C for 5 min. Hydrogen peroxide was detected using a color-developing solution (124 U peroxidase, 80 mg/L 4-aminoantipyrine, and 1.4 g phenol in 50 mM Tris–phosphate buffer, pH 7.2). After stopping the CAT reaction, the reaction mixture was mixed with a color-developing solution, and absorbance was measured at 500 nm. Supplementary Fig. S1 shows the detection system for hydrogen peroxide using the color-developing solution.

### Preparation of decolored SPI and TVP

For the decolorization process, SPI and TVP were incubated with 5–15% hydrogen peroxide for 24 h at 20 °C. After decolorization, hydrogen peroxide solution containing soy proteins was diluted to 5%. To detoxify the hydrogen peroxide, this solution was mixed with 2,500 U CAT and 0.05% AFE1530 and incubated with agitation for 30 min at 20 °C. After foaming and washing, the decolored proteins were lyophilized.

### Color analysis

The colors of SPI, TVP, and meat analogs were measured using a colorimeter (Chroma Meter CM-700d/600d; Konica Minolta, Tokyo, Japan). The color analysis results were expressed using the Commission International de l’Eclairage system and reported as Hunter L* (lightness), a* (redness), and b* (yellowness). The L* value also indicates the whiteness intensity^43^.

### Determination of total phenolic compounds

Total phenolic compounds (free and protein bound) were extracted as described previously^47^. Briefly, protein isolates, previously sifted and dialyzed, were extracted with 80% ethanol for 20 min with continuous shaking at room temperature. This step was repeated three times to collect free phenolic compounds. Next, 2 N NaOH was added directly to the residue and shaken for 90 min at 60 °C. After alkaline treatment, the solution was acidified to pH 2 by adding 6 N HCl and centrifuged to separate the cloudy precipitates. The liberated phenolic acids were extracted three times with ethyl acetate to collect protein-bound phenolic compounds. The obtained extract was mixed and used to quantify the levels of total phenolic compounds using the Folin-Ciocalteu method, as described previously^48^. The concentration of total phenolic compounds was quantified using a standard curve prepared with gallic acid and expressed as mg gallic acid equivalents per 100 g of sample (mg GAE/100 g).

### SDS-PAGE

The effect of hydrogen peroxide on SPI was measured using SDS-PAGE. The freeze-dried proteins were dissolved at a 10% concentration. Protein solutions were centrifuged at 15,000×*g* for 10 min at 20 °C in a high-speed centrifuge. The supernatants were prepared in SDS-PAGE buffer under non-reducing conditions and resolved on a 10–20% separating gel using electrophoresis buffer.

### Degree of hydrolysis

The degree of hydrolysis was determined as previously described^49^. This value was calculated as the ratio (in percentage) of soluble protein in the supernatant after precipitating the protein samples with 0.2 N trichloroacetic acid. The protein content of each sample was determined using the Kjeldahl method (N× 6.25).

### Solubility of H_2_O_2_-treated protein

Protein solubility was determined as previously described method with slight modifications^50^. Freeze-dried proteins were dissolved at 5 mg/mL and then centrifuged at 15,000×*g* for 10 min at 20 °C in a high-speed centrifuge. The supernatant was collected, and its protein content was determined using the Kjeldahl method (N× 6.25). Protein solubility was calculated as the nitrogen solubility index (%) = (protein content of supernatant/amount of proteins added) × 100%.

### Water- and oil-holding capacities

Water- and oil-holding capacities were determined in triplicate according to a previous study with slight modifications^51^. Freeze-dried proteins (0.1 g) were dissolved in 1 g deionized water or dispersed in 1 g olive oil and vortexed for 30 s. After 30 min, the mixture was centrifuged at 2000×*g* at 25 °C for 10 min. The precipitate was weighed, and the water- and oil-holding capacities were expressed as grams of water or oil retained per gram of protein, respectively.

### Foaming capacity and foam stability of hydrogen peroxide-treated protein

Foaming capacity was evaluated as described previously with slight modifications^52,53^. The freeze-dried proteins were dissolved at a concentration of 0.5% (w/v). Foam was formed by homogenizing 50 mL of protein solution at 18,000 rpm for 3 min. The foam and solution were transferred into a 100 mL glass cylinder. The volume of the foam portion was recorded at 0 min to determine the foam capacity. The change in the volume of the foam until 30 min after foaming was monitored to determine foam stability. The following factors were evaluated: foaming capacity (%) = [(VF_0_ − V)/V] × 100 and foam stability (%) = [VF_30_/VF_0_] × 100, where V is the volume of the protein mixture before foam formation, VF_0_ is the volume of foam immediately after homogenization, and VF_30_ is the volume of foam after 30 min.

### Emulsifying activity and stability index of hydrogen peroxide-treated protein

The emulsifying properties (emulsifying activity and stability) were determined as described previously with slight modifications^52,53^. Olive oil (10 mL) and 1% protein solution (30 mL) were mixed. The mixture was homogenized at 10,000 rpm for 2 min. An aliquot of the emulsion (50 μL) was pipetted from the bottom of the container at 0 and 10 min after homogenization and mixed with 5 mL 0.1% SDS solution. The absorbance of the diluted solutions was measured at 500 nm using a spectrophotometer. The absorbance values measured immediately (A_0_) and at 10 min (A_10_) after emulsion formation were used to calculate the emulsifying activity index and emulsion stability index as follows: emulsifying activity index (m^2^/g) = (2 × 2.303 × A_0_)/(F × protein weight), emulsion stability index (%) = (A_10_ × *Δ*t)/(A_0_ − A_10_), where F is the oil volume fraction of 0.25, A_0_ is the absorbance immediately after homogenization, A_10_ is the absorbance at 10 min after homogenization, and *Δ*t = 10 min.

### In vitro digestion of decolored proteins

The in vitro digestion protocol used in this study was based on the INFOGEST digestion method^54^. We prepared 1.25 × electrolyte stock solutions for each digestion phase and measured the enzyme activities before the experiments. Samples and stock solutions were preheated to 37 °C; all digestion processes were performed at 37 °C. All phase samples were collected and boiled at 100 °C for 5 min. Each amino acid was quantified using an Agilent 1100 HPLC (Agilent Technologies, Santa Clara, CA, USA) equipped with a fluorescence detector (excitation 230 nm and emission 450 PMT 9 or excitation 266 nm and emission 305 nm PMT 7). The supernatant was treated with *o*-phthalaldehyde and 9-fluorenylmethyl chloroformate and separated on a Zorbax Eclipse-AAA column (4.6 × 150 mm, Agilent) with a gradient of 20 mM Na_2_HPO_4_ buffer (pH 8.2) and methanol:acetonitrile:H_2_O = 45:45:10 for 20 min at a flow rate of 0.65 mL/min.

In the oral phase, 5 mL of simulated salivary fluids (pH 7.0) was added to a beaker containing 5 g of non-treated and hydrogen peroxide-treated proteins. The mixtures were stirred for 2 min to mimic the agitation of foods within the human mouth. Amylase activity was 75 U/mL in the final oral mixture.

In the gastric phase, simulated gastric fluids were added to the sample from the oral phase to a final volume ratio of 1:1, after which the mixture was rapidly adjusted to pH 3.0. Pepsin activity was 2,000 U/mL in the final stomach mixture. Protein digestion in the stomach was performed at pH 3.0 for 2 h.

In the intestinal phase, 20 mL of simulated intestinal fluids was added to the sample from the stomach phase, and the resulting mixture was adjusted to pH 7.0. In the final small intestine mixture, the concentration of bile salts was 10 mM, trypsin activity was 100 U/mL, and lipase activity was 2,000 U/mL. The mixture was stirred in a water bath for 2 h to mimic the intestinal conditions.

### Preparation of decolored TVP-based foods

Non-treated or decolored TVP was mixed with 2.0% methylcellulose as the final concentration. Next, 10 g of water, 2 g of olive oil, and 2.5 g of PI were added to 25 g of each TVP matrix. The samples were blended for 60 s using a hand blender. The TVP matrix was molded (60 mm × 40 mm area × 25 mm height) and then cooked at 150 °C for 15 min, and then cooled to room temperature (20–25 °C) before further analysis.

### Texture profile analysis of patties

Texture profile analysis was performed using a COMPAC-100II (Sun Scientific Co., Ltd., Tokyo, Japan) equipped with a cylindrical probe with an area of 31.4 mm^55^. After grilling, meat analogs were prepared for analysis and cut to a length of 15 mm to obtain homogeneous extrudates. The diameter of each extrudate was approximately 20 mm. A double-compression cycle was performed at 1 mm/s until a recorded deformation of 50% was achieved. The following parameters were evaluated: hardness, maximum force recorded during the first compression; cohesiveness, area of work during the second compression divided by the area of work during the first compression; springiness, distance recorded during the second compression divided by the distance of the first compression; and chewiness, hardness × cohesiveness × springiness.

### Cooking loss

The cooking method and conditions were determined based on a previously described method^56^. The patties were cooked at 150 °C for 10 min or until the temperature at the center of the patties reached 80 °C. The samples were cooled to room temperature. Cooking loss was calculated as the percentage weight difference between the patty before cooking and after cooking using the following formula: cooking loss (%) = [(W_1_ − W_2_)/W_1_] × 100, where W_1_ and W_2_ are the weights of the patty before and after grilling (g), respectively.

## Supplementary Information

Supplementary Figures.

## Data Availability

All data generated or analyzed during this study are included in this published article and its supplementary information files.
