# Supplementary material for: Decolorization and detoxication of plant-based proteins using hydrogen peroxide and catalase
Source: Sci Rep. 2022 Dec 27;12:22432. doi: 10.1038/s41598-022-26883-8 (PMC9794787; doi:10.1038/s41598-022-26883-8)
Supplement: Supplementary file 1 — Supplementary Figures. [file 41598_2022_26883_MOESM1_ESM.docx]

**Decolorization and detoxication of plant-based proteins using hydrogen peroxide and catalase**

Kiyota Sakai, Masamichi Okada, Shotaro Yamaguchi

**Supplementary Materials**


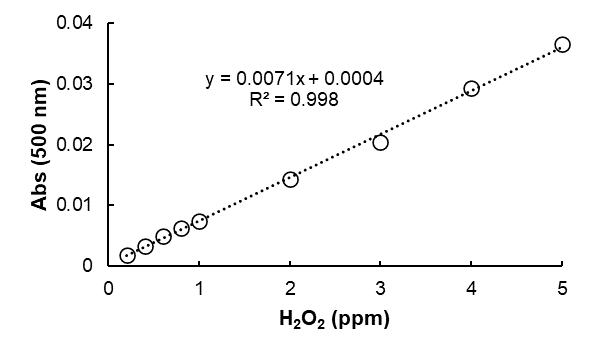


**Figure S1**: Standard curves of hydrogen peroxide detected using the peroxidase and 4AA-phenol method.


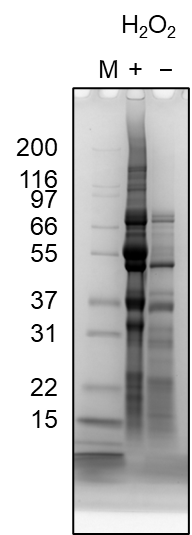


**Figure S2. SDS-PAGE analysis of decolored soy-based protein isolate.**

Lane 1, decolored protein; Lane 2, non-treated protein; Lane M, protein molecular mass makers.
